# Robust model selection using the out-of-bag bootstrap in linear regression

**DOI:** 10.1038/s41598-022-14398-1

**Published:** 2022-06-29

**Authors:** Fazli Rabbi, Alamgir Khalil, Ilyas Khan, Muqrin A. Almuqrin, Umair Khalil, Mulugeta Andualem

**Affiliations:** 1 Department of Statistics, University of Peshawar, Peshawar, Pakistan; 2 Department of Mathematics, College of Science Al-Zulfi, Majmaah University, Al-Majmaah, 11952 Saudi Arabia; 3 Department of Mathematics, College of Science in Zulfi, Majmaah University, Al-Majmaah, 11952 Saudi Arabia; 4 Department of Statistics Abdul Wali, Khan University, Mardan, Pakistan; 5 Department of Mathematics, Bonga University, Bonga, Ethiopia

**Keywords:** Mathematics and computing, Statistics

## Abstract

Outlying observations have a large influence on the linear model selection process. In this article, we present a novel approach to robust model selection in linear regression to accommodate the situations where outliers are present in the data. The model selection criterion is based on two components, the robust conditional expected prediction loss, and a robust goodness-of-fit with a penalty term. We estimate the conditional expected prediction loss by using the out-of-bag stratified bootstrap approach. In the presence of outliers, the stratified bootstrap ensures that we obtain bootstrap samples that are similar to the original sample data. Furthermore, to control the undue effect of outliers, we use the robust MM-estimator and a bounded loss function in the proposed criterion. Specifically, we observe that instead of minimizing the penalized loss function or the conditional expected prediction loss separately, it is better to minimize them simultaneously. The simulation and real-data based studies confirm the consistent and satisfactory behavior of our bootstrap model selection procedure in the presence of response outliers and covariate outliers.

## Introduction

A variety of models are used in statistical modeling. Often the focus is to identify the single best model, which describes the data well while being parsimonious. The model selection procedure involves fitting a set of competing models and then selecting the best model by comparing the values of their goodness-of-fit statistics, their prediction loss, or both of these two. Several studies on model selection procedures have concluded that these methods depend on maximum likelihood-type or least squares approaches^1–6^ and are possibly affected by the presence of outlying observations in the data. Robust model selection methods aim to work well in situations when some of the observations are outliers and/or the error distribution is not normal. Several robust model selection procedures have been proposed in the literature. To cope with these problems in model selection, different approaches are proposed. Some of them are based on robust modifications of well-known standard criteria such as Akaike information criterion or Mallows’ $C_{p}$ criterion, or on various resampling techniques, like bootstrap or cross-validation^7–18^. The main objective of this research work is to propose a modified version of^19^ for model selection in the presence of outliers.

Suppose that we have a column vector of ***n*** responses $Y{\mathbf{ = (y}}_{{\mathbf{1}}} {\mathbf{,y}}_{{\mathbf{2}}} {\mathbf{,}}...{\mathbf{,y}}_{{\mathbf{n}}} {\mathbf{)}}^{{\mathbf{T}}} {\mathbf{,}}$ and $X$ is an ${\mathbf{n }} \times {\mathbf{ p}}$ design matrix. Let ${{\varvec{\upalpha}}}$ denote any subset of size ${\mathbf{p}}_{{{\varvec{\upalpha}}}}$ from ${\mathbf{\{ }}\,{\mathbf{1}}\,{\mathbf{,}}\,\,{\mathbf{2}}\,{\mathbf{,}}\,{\mathbf{.}}\,\,{\mathbf{.}}\,\,{\mathbf{.}}\,\,{\mathbf{,}}\,\,{\mathbf{p\} }}$, and let ${\mathbf{X}}_{{{\varvec{\upalpha}}}}$ is an ${\mathbf{n \times p}}_{{{\varvec{\upalpha}}}}$ matrix. Let ${\mathbf{x}}_{{{\mathbf{\alpha i}}}}^{{\mathbf{T}}}$ denote the ${\mathbf{i}}$th row vector of the design matrix ${\mathbf{X}}_{{{\varvec{\upalpha}}}}$. Then the linear regression model corresponding to model α is given by

1 $${\mathbf{y}}_{{\mathbf{i}}} {\mathbf{ = x}}_{{{\mathbf{\alpha i}}}}^{{\mathbf{T}}} {{\varvec{\upbeta}}}_{{{\varvec{\upalpha}}}} {\mathbf{ + \varepsilon }}_{{{\mathbf{\alpha i}}}} {\mathbf{,}}\quad {\mathbf{i}}\,\,{\mathbf{ = }}\,\,{\mathbf{1,}}\,\,{\mathbf{2,}}\,\,...{\mathbf{,}}\,{\mathbf{n}}$$

where ${\text{X}}_{\alpha }$ and ${{\varvec{\upvarepsilon}}}_{{{{\varvec{\upalpha}}}i}} = {\mathbf{(\varepsilon }}_{{\alpha {\mathbf{1}}}} {\mathbf{,\varepsilon }}_{{\alpha {\mathbf{2}}}} {\mathbf{,}}...{\mathbf{,\varepsilon }}_{{\alpha {\mathbf{n}}}} {\mathbf{)}}^{{\mathbf{T}}}$ are independent, and the errors ${{\varvec{\upvarepsilon}}}_{{{\mathbf{\alpha i}}}}$ are assumed to have location 0 and scale 1, ${{\varvec{\upbeta}}}_{{{\varvec{\upalpha}}}}$ is an unknown ${\mathbf{p}}_{{{\varvec{\upalpha}}}}$-vector of regression coefficients. Let ${\rm A}$ represent a collection of candidate models. The interest here is to select a model ${{\varvec{\upalpha}}}$ from ${\rm A}$ based on the specified properties of the corresponding fit. To fit the linear regression model, the MM-estimator of^20^ was adopted, which combines excellent robustness properties along with high efficiency in the absence of outliers in the data. In model selection, three aspects are generally considered i.e., specifying an estimator, fitting models by using the specified estimator and finally, the fitted models are compared. Furthermore, for each of the models ${{\varvec{\upalpha}}}$ the approach can be extended by considering various types of estimators like LS-estimator, M-estimator, and MM-estimator etc. The models are indexed by ${{\varvec{\upalpha}}} \in {\rm A}$ and estimate ${{\varvec{\upbeta}}}_{{{\varvec{\upalpha}}}}$ by the estimator ${\hat{\mathbf{\beta }}}_{{{\varvec{\upalpha}}}}$.

The following two minimal requirements for a good model are discussed by^19^:

(i) it has the capability to fit the sample data ***y*** and ***X*** reasonably well, and
(ii) it has the ability to predict future observations with great accuracy.

The ability of a model to fit the sample data ***y*** and ***X*** is measured by applying a penalized loss function and the expected prediction loss is used to measure the ability to predict future observations. It has been found in the literature that bootstrapping a robust estimator encounters some difficulties in the presence of outliers. For robust regression, an ***m***-out-of-***n*** paired bootstrap approach is proposed by^12^. Their study findings revealed that implementing the bootstrap procedure directly to a data set containing outliers, generally, fails due to two reasons: (1) The use of $\rho (x) = x^{2}$, which is non-robust against outliers, and (2) the bootstrap samples may contain a high proportion of outliers as compared to the original data set. Müller and Welsh^19^ addressed both of these issues by using stratified bootstrap with appropriate choice of $\rho (.)$ in the presence of outliers. Their approach ensured that one can obtain bootstrap samples that are similar to the sample data. According to their approach, bootstrap samples are constructed in such a manner that the residuals distribution for each bootstrap sample will reflect the relatively same residuals distribution observed in the original data. Their strategy seems to solve the issue well in practice.

Our objective in this paper is to pursue the investigation in^19^ and make some refinements, by utilizing the concept of out-of-bag bootstrap to develop a robust model selection criterion which deals with outliers and heavy tailed error distributions. The out-of-bag (OOB) observations are those which are not part of the bootstrap sample. These OOB observations can be used for estimating the prediction error, yielding the so-called OOB error. This type of error is often claimed to be an unbiased estimator for the true error rate^21,22^.

The rest of the paper is organized as follows: We discuss the existing robust model selection criteria in “Robust model selection criteria” section. Section “The proposed robust model selection criterion” describes a proposed robust model selection criterion. We show the performance of our modified robust criterion via simulation studies in “Simulation studies” section. We present a data examplein “Data example (Stack loss data)” section and conclude with a short discussion in “Conclusion” section.

## Robust model selection criteria

In this section, we discuss the existing robust model selection criteria based on robust expected prediction loss. Consider a vector of ***n*** responses ${\mathbf{y}}_{{\mathbf{i}}} {\mathbf{ = (y}}_{{\mathbf{1}}} {\mathbf{,y}}_{{\mathbf{2}}} {\mathbf{,}}...{\mathbf{,y}}_{{\mathbf{n}}} {\mathbf{)}}^{{\mathbf{T}}}$ and the design matrix ${\mathbf{X = (x}}_{{\mathbf{1}}} {\mathbf{,x}}_{{\mathbf{2}}} {\mathbf{,}}...{\mathbf{,x}}_{{\mathbf{n}}} {\mathbf{)}}^{{\mathbf{T}}}$. The conditional expected prediction loss of a model α for a given non- negative loss function $\rho (.)$ is calculated by

2 $${\mathbf{M}}^{{{\mathbf{PE}}}} {\mathbf{(\alpha ) = }}\frac{{{{\varvec{\upsigma}}}^{{\mathbf{2}}} }}{{\mathbf{n}}}{\mathbf{E}}\left[ {\sum\limits_{{{\mathbf{i = 1}}}}^{{\mathbf{n}}} {{\varvec{\uprho}}} \left\{ {\frac{{{\mathbf{z}}_{{\mathbf{i}}} {\mathbf{ - x}}_{{{\mathbf{\alpha i}}}}^{{\mathbf{T}}} {\hat{\mathbf{\beta }}}_{{{\varvec{\upalpha}}}}^{{}} }}{{{\varvec{\upsigma}}}}} \right\}\left| {{\mathbf{y,X}}} \right.} \right]$$

where ${\hat{\mathbf{\beta }}}_{{{\varvec{\upalpha}}}}$ is the estimator of ${{\varvec{\upbeta}}}_{{{\varvec{\upalpha}}}}$, ${\mathbf{z = (z}}_{{\mathbf{1}}} {\mathbf{,z}}_{{\mathbf{2}}} {\mathbf{,}}...{\mathbf{,z}}_{{\mathbf{n}}} {\mathbf{)}}^{{\mathbf{T}}}$ is a vector of future responses at X, independent of y, and $\sigma$ is the measure of spread for a given data. Initially, this type of prediction loss was introduced by^5^ as a model selection criterion by using a loss function $\rho (x) = \frac{{x^{2} }}{2}$ in the least squares regression.

To select a model $\alpha$ from a set ${\rm A}$,^19^ proposed the following criterion function

3 $${\mathbf{M}}_{{\mathbf{n}}} {\mathbf{(\alpha ) = }}\frac{{{{\varvec{\upsigma}}}^{{\mathbf{2}}} }}{{\mathbf{n}}}\left[ {\sum\limits_{{{\mathbf{i = 1}}}}^{{\mathbf{n}}} {{{\varvec{\uprho}}}\left\{ {\frac{{{\mathbf{y}}_{{\mathbf{i}}} {\mathbf{ - x}}_{{{\mathbf{\alpha i}}}}^{{\mathbf{T}}} {\hat{\mathbf{\beta }}}_{{{\varvec{\upalpha}}}} }}{{{\varvec{\upsigma}}}}} \right\}} {\mathbf{ + \delta (n)p}}_{{{\varvec{\upalpha}}}} {\mathbf{ + E}}\sum\limits_{{{\mathbf{i = 1}}}}^{{\mathbf{n}}} {{{\varvec{\uprho}}}\left\{ {\frac{{{\mathbf{z}}_{{\mathbf{i}}} {\mathbf{ - x}}_{{{\mathbf{\alpha i}}}}^{{\mathbf{T}}} {\hat{\mathbf{\beta }}}_{{{\varvec{\upalpha}}}} }}{{{\varvec{\upsigma}}}}} \right\}\left| {{\mathbf{y,X}}} \right.} } \right]$$

Following^5,19^ estimated the unknown distribution of the data by usingan *m*-out-of-*n* stratified bootstrap procedure, whereas the penalized in-sample term in (3) is estimated directly. The estimated selection criteria functions are given by

4 $${\mathbf{M}}_{{{\mathbf{m,n}}}}^{{{\mathbf{PE}}}} {\mathbf{(\alpha ) = }}\frac{{{\hat{\mathbf{\sigma }}}^{{\mathbf{2}}} }}{{\mathbf{n}}}{\mathbf{E}}_{{\mathbf{*}}} \left[ {\sum\limits_{{{\mathbf{i = 1}}}}^{{\mathbf{n}}} {{\varvec{\uprho}}} \left\{ {\frac{{{\mathbf{y}}_{{\mathbf{i}}} {\mathbf{ - x}}_{{{\mathbf{\alpha i}}}}^{{\mathbf{T}}} {\hat{\mathbf{\beta }}}_{{{\mathbf{\alpha ,m}}}}^{{\mathbf{*}}} }}{{{\hat{\mathbf{\sigma }}}}}} \right\}} \right]$$

5 $${\mathbf{M}}_{{{\mathbf{m,n}}}}^{{{\mathbf{PPE}}}} {\mathbf{(\alpha ) = }}\frac{{{\hat{\mathbf{\sigma }}}^{{\mathbf{2}}} }}{{\mathbf{n}}}\left[ {\sum\limits_{{{\mathbf{i = 1}}}}^{{\mathbf{n}}} {{{\varvec{\uprho}}}\left\{ {\frac{{{\mathbf{y}}_{{\mathbf{i}}} {\mathbf{ - x}}_{{{\mathbf{\alpha i}}}}^{{\mathbf{T}}} {\hat{\mathbf{\beta }}}_{{{\varvec{\upalpha}}}} }}{{{\hat{\mathbf{\sigma }}}}}} \right\}} {\mathbf{ + \delta (n)p}}_{{{\varvec{\upalpha}}}} {\mathbf{ + E}}_{{\mathbf{*}}} \sum\limits_{{{\mathbf{i = 1}}}}^{{\mathbf{n}}} {{{\varvec{\uprho}}}\left\{ {\frac{{{\mathbf{y}}_{{\mathbf{i}}} {\mathbf{ - x}}_{{{\mathbf{\alpha i}}}}^{{\mathbf{T}}} {\hat{\mathbf{\beta }}}_{{{\mathbf{\alpha ,m}}}}^{{\mathbf{*}}} }}{{{\hat{\mathbf{\sigma }}}}}} \right\}} } \right]$$

where ${\hat{\mathbf{\beta }}}_{{{\mathbf{\alpha ,m}}}}^{{\mathbf{*}}}$ is the bootstrap estimate of ${\hat{\mathbf{\beta }}}_{{{\varvec{\upalpha}}}}^{{}}$, $E_{*}$ denotes expectation with respect to the bootstrap distribution and ***m*** is the number of distinct observations in the bootstrap sample which satisfies the conditions given by

$$m \to \infty \;{\text{and}}\;\frac{m}{\sqrt n } \to 0\;{\text{as}}\;n \to \infty .$$

The criterion function in (4) was modified by^18^ using the following steps:

(i) calculate and order the residuals,
(ii) set the number of strata S at between 3 and 8 depending on the sample size *n*,
(iii) set stratum boundaries of the residuals,
(iv) allocate observations into different strata so that observations in the extreme tail are kept in lower or upper tail strata and other strata comprising the remaining observations,
(v) sample rows of (y,X) independently with replacement from each stratum so that total bootstrap sample of size is $m\,( \le n),$
(vi) construct the estimator $\hat{\beta }_{\alpha ,m}^{*}$ from data obtained in step (v),
(vii) calculate the criterion function ${\mathbf{M}}_{{{\mathbf{m,n}}}}^{{{\mathbf{PE*}}}} {\mathbf{(\alpha )}}$ from *n-m* observations i.e., ***m*** observations used to obtain ${\hat{\mathbf{\beta }}}_{{{\mathbf{\alpha ,m}}}}^{{\mathbf{*}}}$ are not included when calculating ${\mathbf{M}}_{{{\mathbf{m,n}}}}^{{{\mathbf{PE*}}}} {\mathbf{(\alpha )}}$,
(viii) repeat the steps (vi) and (vii) ***K*** independent times and then estimate the modified robust expected prediction loss by
  6 $${\mathbf{M}}_{{{\mathbf{m,n}}}}^{{{\mathbf{PE*}}}} {\mathbf{(\alpha ) = }}\frac{{{\hat{\mathbf{\sigma }}}^{{\mathbf{2}}} }}{{\mathbf{n}}}\left[ {{\mathbf{E}}_{{\mathbf{*}}} \sum\limits_{{{\mathbf{i = 1}}}}^{{{\mathbf{n - m}}}} {{{\varvec{\uprho}}}\left\{ {\frac{{{\mathbf{y}}_{{{\mathbf{i}}\left[ {{\mathbf{ - m}}} \right]}} {\mathbf{ - x}}_{{{\mathbf{\alpha i[ - m]}}}}^{{\mathbf{T}}} {\hat{\mathbf{\beta }}}_{{{\mathbf{\alpha ,m}}}}^{{\mathbf{*}}} }}{{{\hat{\mathbf{\sigma }}}}}} \right\}} } \right]$$
  where ${\hat{\mathbf{\beta }}}_{{{\mathbf{\alpha ,m}}}}^{{\mathbf{*}}}$ is the bootstrap estimate of ${\hat{\mathbf{\beta }}}_{{{\varvec{\upalpha}}}}^{{}}$, $E_{*}$ denotes expectation with respect to the bootstrap distribution and ***m*** is the number of distinct observations in the bootstrap sample used to obtain $\hat{\beta }_{\alpha ,m}^{*}$ and [-m] means that the ***m*** observations are excluded from total observations when calculating $M_{m,n}^{PE*} (\alpha )$. Here the focus is on the model $\alpha \in A$ that minimizes $M_{m,n}^{PE} (\alpha )$, $M_{m,n}^{PPE} (\alpha )$ or ${\mathbf{M}}_{{{\mathbf{m,n}}}}^{{{\mathbf{PE*}}}} {\mathbf{(\alpha )}}$ i.e.
  7 $${\overline{\mathbf{\alpha }}}_{{{\mathbf{m,n}}}} = \mathop {{\mathbf{arg}}\,{\mathbf{min}}}\limits_{{\alpha \in {\rm A}}} {\mathbf{ M}}_{{{\mathbf{m,n}}}}^{{{\mathbf{PE}}}} {\mathbf{(\alpha )}}$$
  8 $${\tilde{\mathbf{\alpha }}}_{{{\mathbf{m,n}}}} {\mathbf{ = }}\mathop {{\mathbf{arg}}\,\,{\mathbf{min}}}\limits_{{\alpha \in {\rm A}}} {\mathbf{ M}}_{{{\mathbf{m,n}}}}^{{{\mathbf{PPE}}}} {\mathbf{(\alpha }})$$
  9 $${\hat{\mathbf{\alpha }}}_{{{\mathbf{m,n}}}} = \mathop {\arg \min }\limits_{{\alpha \in {\rm A}}} \, {\mathbf{M}}_{{{\mathbf{m,n}}}}^{{{\mathbf{PE*}}}} {\mathbf{(\alpha )}}$$

## The proposed robust model selection criterion

In this section, we propose a robust model selection procedure based on two components, a robust penalized loss function, and a modified robust expected prediction loss.

We estimate the penalized in-sample term in the criterion function by

10 $${\mathbf{M}}_{{\mathbf{n}}}^{{\mathbf{P}}} {\mathbf{(\alpha ) = }}\frac{{{\hat{\mathbf{\sigma }}}^{{\mathbf{2}}} }}{{\mathbf{n}}}\left[ {\sum\limits_{{{\mathbf{i = 1}}}}^{{\mathbf{n}}} {{{\varvec{\uprho}}}\left\{ {\frac{{{\mathbf{y}}_{{\mathbf{i}}} {\mathbf{ - x}}_{{{\mathbf{\alpha i}}}}^{{\mathbf{T}}} \widehat{{{{\varvec{\upbeta}}}_{{{\varvec{\upalpha}}}} }}}}{{{\hat{\mathbf{\sigma }}}}}} \right\}} {\mathbf{ + \delta (n)p}}_{{{\varvec{\upalpha}}}} } \right]$$

where ${\mathbf{\delta (n)}}$ denotes a function of sample size ***n***. The two restrictions on function ${\mathbf{\delta (n)}}$ are that ${\mathbf{\delta (n)}} \to \infty$ and $\frac{{{\mathbf{\delta (n)}}}}{{\mathbf{n}}} \to 0$ as $n \to \infty$. The two restrictions on δ (n) are imposed to penalize complexity, which expresses a preference for smaller and simpler models. These conditions are satisfied by the choice ${\mathbf{\delta (n)}} = \,\log \,(n)$. We combine (6) and (10) to estimate the robust criterion function by

11 $${\mathbf{M}}_{{{\mathbf{m,n}}}}^{{{\mathbf{PPE*}}}} {\mathbf{(\alpha ) = }}\frac{{{\hat{\mathbf{\sigma }}}^{{\mathbf{2}}} }}{{\mathbf{n}}}\left[ {\sum\limits_{{{\mathbf{i = 1}}}}^{{\mathbf{n}}} {{{\varvec{\uprho}}}\left\{ {\frac{{{\mathbf{y}}_{{\mathbf{i}}} - {\mathbf{x}}_{{{\mathbf{\alpha i}}}}^{{\mathbf{T}}} {\hat{\mathbf{\beta }}}_{{{\varvec{\upalpha}}}} }}{{{\hat{\mathbf{\sigma }}}}}} \right\}} {\mathbf{ + \delta (n)p}}_{{{\varvec{\upalpha}}}} {\mathbf{ + E}}_{{\mathbf{*}}} \sum\limits_{{{\mathbf{i = 1}}}}^{{{\mathbf{n - m}}}} {{{\varvec{\uprho}}}\left\{ {\frac{{{\mathbf{y}}_{{{\mathbf{i}}\left[ {{\mathbf{ - m}}} \right]}} - {\mathbf{x}}_{{{\mathbf{\alpha i[ - m]}}}}^{{\mathbf{T}}} {\hat{\mathbf{\beta }}}_{{{\mathbf{\alpha ,m}}}}^{{\mathbf{*}}} }}{{{\hat{\mathbf{\sigma }}}}}} \right\}} } \right]$$

where ${\hat{\mathbf{\beta }}}_{{{\mathbf{\alpha ,m}}}}^{{\mathbf{*}}}$ is the bootstrap estimate of ${\hat{\mathbf{\beta }}}_{{{\varvec{\upalpha}}}}^{{}}$, $E_{*}$ denotes expectation with respect to the bootstrap distribution and ***m*** is the number of distinct observations in the bootstrap sample. An important issue is “how large should the number of bootstrap replications K in our proposed criterion. There is no hard and fast rule for the number of bootstrap replications. However, for estimation of standard error, it is usually in the range of 25–250^23^. The first term in criterion function (11) measures the relationship between the observed sample data ***y*** and ***X***; the second term penalizes complexity (i.e., preference for smaller models), while the ability to predict future observations is measured by the last term. To use (11), we have to specify $\rho (.)$ and $\sigma$. The robustness viewpoint is adopted for the purpose of fitting the core of the data and predicting core observations, rather than fitting and predicting the tails having atypical observations. So a bounded $\rho$ function is selected. Here, trimming is preferred, so that for sufficiently large |x| the $\rho (x)$ function is constant.

As in^11,14,18,19^, the simplest $\rho$ function is given by

12 $${\mathbf{\rho (x) = min(x}}^{{\mathbf{2}}} {\mathbf{,b}}^{{\mathbf{2}}} {\mathbf{)}}$$

which is quadratic near the origin and becomes constant when it is away from the origin. As in^19^, we use ***b*** = 2. To measure spread $\sigma ,$ we use the full model $\alpha_{{\varvec{f}}}$, because for residuals spread, a large model can produce a valid measure. For simplicity, we measure $\sigma$ by the median absolute deviation (MAD) from the median multiplied by 1.483 and is given by

$${\hat{\mathbf{\sigma }}} = 1.483\mathop {med}\limits_{1 \le i \le n} \left| {e_{i} - \mathop {med}\limits_{1 \le j \le n} (e_{j} )} \right|$$

where ${\mathbf{ e}}_{{\mathbf{i}}} {\mathbf{ = y}}_{{\mathbf{i}}} - {\mathbf{x}}_{{{{\varvec{\upalpha}}}_{{\mathbf{f}}} {\mathbf{i}}}}^{{\mathbf{T}}} {\hat{\mathbf{\beta }}}_{{{{\varvec{\upalpha}}}_{{\mathbf{f}}} }} {\mathbf{,}}\;{\mathbf{ e}}_{{\mathbf{j}}} {\mathbf{ = y}}_{{\mathbf{j}}} -_{{{{\varvec{\upalpha}}}_{{\mathbf{f}}} {\mathbf{j}}}}^{{\mathbf{T}}} {\hat{\mathbf{\beta }}}_{{{{\varvec{\upalpha}}}_{{\mathbf{f}}} }}$ and $\hat{\beta }_{\alpha }^{{}}$ is the estimator for ${{\varvec{\upbeta}}}_{{{\varvec{\upalpha}}}}$.

Among the models being considered, we select a model $\alpha \in A$ that minimizes ${\mathbf{M}}_{{{\mathbf{m,n}}}}^{{{\mathbf{PPE*}}}} {\mathbf{(\alpha )}}$, i.e.

13 $${\hat{\mathbf{\alpha }}}_{{{\mathbf{m,n}}}}^{{\mathbf{*}}} = \mathop {\arg \min }\limits_{{\alpha \in {\rm A}}} {\mathbf{M}}_{{{\mathbf{m,n}}}}^{{{\mathbf{PPE*}}}} {\mathbf{(\alpha )}}$$

The optimal ***m*** depends on the true model. As in^14,19^, one should use ***n*/4** ≤  ***m* ≤ *n*/2** for moderate ***n*** (50 ≤  ***n*** ≤ 200). If ***n*** is small, ***m*** is small and the parameter estimators do not converge for some bootstrap samples; but if ***n*** is large, ***m*** may be smaller than a fourth of ***n***. Choosing the number of strata **S** at between 3 and 8, depending on the sample size ***n***^24^.

The penalized loss function in the proposed criterion function, given in (10), is just like a robust version of AIC proposed by^25,26^. But the main difference is due to the $\rho$ function and the estimator in our criterion. The penalized in-sample term in (11) is similar to the robust version of^3^. Furthermore, for $\rho (x) = (x^{2} ),$ the penalized in-sample term was reduced to^3^ criterion.

## Simulation studies

To assess and compare the finite sample performance of our proposed method with the existent model selection methods, we carried out two simulation studies, that is, one for contamination free dataset in a simulation setting 1 and the other for the contaminated data set in a simulation setting 2.

### Simulation setting 1

The finite-sample performance of our proposed criterion is compared with existing model selection procedures via real dataset and simulated data set.

#### The Gunst and Mason data

To compare the finite sample performance of our proposed method with the existent model selection methods through the real dataset, we use the following regression mode

$${\mathbf{Y}}_{{\mathbf{i}}} {\mathbf{ = \beta }}_{{\mathbf{0}}} {\mathbf{X}}_{{{\mathbf{i0}}}} {\mathbf{ + \beta }}_{{\mathbf{1}}} {\mathbf{X}}_{{{\mathbf{i1}}}} {\mathbf{ + \beta }}_{{\mathbf{2}}} {\mathbf{X}}_{{{\mathbf{i2}}}} {\mathbf{ + \beta }}_{{\mathbf{3}}} {\mathbf{X}}_{{{\mathbf{i3}}}} {\mathbf{ + \beta }}_{{\mathbf{4}}} {\mathbf{X}}_{{{\mathbf{i4}}}} {\mathbf{ + u}}_{{\mathbf{i}}} {\mathbf{,}}\quad {\mathbf{i = 1,}}\,{\mathbf{2,}}\,...{\mathbf{,}}\,{\mathbf{40}}$$

where $$u_{i}$$ are iid standard normal errors;$$X_{0}$$ is the column of 1’s; and the values of $$X_{1} ,X_{2} ,X_{3}$$ and $$X_{4}$$ are taken from the solid waste data of^27^, as in^5,12,13,18,19^. We compare the estimator $$\hat{\alpha }^{*}_{m,n}$$ [expressed in (13)], with $$\overline{\alpha }_{m,n}$$ [expressed in (7)], $$\tilde{\alpha }_{m,n}$$ [expressed in (8)], $${\hat{\mathbf{\alpha }}}_{{{\mathbf{m,n}}}}$$ [expressed in (9)] and robust BIC $$\overset{\lower0.5em\hbox{$\smash{\scriptscriptstyle\smile}$}}{\alpha }_{n}$$ [expressed in (14)],

14 $${\mathbf{\overset{\lower0.5em\hbox{$\smash{\scriptscriptstyle\smile}$}}{\alpha } }}_{{\mathbf{n}}}^{{}} = \mathop {\arg \min }\limits_{{\alpha \in {\rm A}}} \, {\mathbf{M}}_{{\mathbf{n}}}^{{\mathbf{P}}} {\mathbf{(\alpha )}}$$

In the zero contamination case, the least squares estimator is used to fit the regression models. The penalty term $$\delta (n) = \log (n)$$ is used in all simulations. The estimated selection probabilities for $$\hat{\alpha }_{m,n}^{*}$$,$$\overline{\alpha }_{m,n}$$,$$\tilde{\alpha }_{m,n}$$ and $$\overset{\lower0.5em\hbox{$\smash{\scriptscriptstyle\smile}$}}{\alpha }_{n}$$ based on the LS estimator and $${\mathbf{\rho (x) = x}}^{{\mathbf{2}}}$$ are mentioned in Table 1, whereas the estimated selection probabilities based on the LS estimator and $${\mathbf{\rho (x) = min(x}}^{{\mathbf{2}}} {\mathbf{,b}}^{{\mathbf{2}}} {\mathbf{)}}$$ are given in Table 2. The results given in Tables 1 and 2 are based on ***L*** = 1000 simulations and ***K*** = 100 bootstrap samples for ***m*** = 15, 20, 25.

**Table 1:** Estimated selection probabilities of $\overline{\alpha }_{m,n}$ , $\tilde{\alpha }_{m,n}$, ${\hat{\mathbf{\alpha }}}_{{{\mathbf{m,n}}}}$ and $\hat{\alpha }_{m,n}^{*}$ based on the least squares estimator and $\rho (x^{2} )$.

True $$\beta ^{T}$$	Model	m = 15				m = 20				m = 25				$$\overset{\lower0.5em\hbox{$\smash{\scriptscriptstyle\smile}$}}{\alpha }_{40}$$
		$\overline{\alpha }_{15,40}$	${\hat{\mathbf{\alpha }}}_{{15{\mathbf{,}}40}}$	$\tilde{\alpha }_{15,40}$	$\hat{\alpha }_{15,40}^{*}$	$\overline{\alpha }_{20,40}$	${\hat{\mathbf{\alpha }}}_{{20{\mathbf{,}}40}}$	$\tilde{\alpha }_{20,40}$	$\hat{\alpha }_{20,40}^{*}$	$\overline{\alpha }_{25,40}$	${\hat{\mathbf{\alpha }}}_{{25{\mathbf{,}}40}}$	$\tilde{\alpha }_{25,40}$	$\hat{\alpha }_{25,40}^{*}$	
**(2,0,0,4,0)**	1,4*	**0.943**	**0.972**	**0.928**	**0.958**	**0.875**	**0.943**	**0.876**	**0.925**	**0.770**	**0.903**	**0.814**	**0.896**	**0.835**
	1,4,5	0.010	0.006	0.013	0.009	0.024	0.014	0.030	0.018	0.042	0.023	0.038	0.027	0.046
	1,3,4	0.019	0.010	0.021	0.011	0.050	0.014	0.044	0.023	0.100	0.038	0.075	0.039	0.046
	1,2,4	0.028	0.012	0.036	0.022	0.046	0.029	0.046	0.032	0.069	0.034	0.060	0.036	0.057
	1,3,4,5	0.000	0.000	0.001	0.000	0.001	0.000	0.001	0.001	0.005	0.001	0.004	0.001	0.004
	1,2,4,5	0.000	0.000	0.000	0.000	0.001	0.000	0.001	0.001	0.004	0.000	0.005	0.000	0.009
	1,2,3,4	0.000	0.000	0.001	0.000	0.003	0.000	0.002	0.000	0.008	0.001	0.004	0.001	0.003
	1,2,3,4,5	0.000	0.000	0.000	0.000	0.000	0.000	0.000	0.000	0.002	0.000	0.000	0.000	0.000
**(2,0,0,4,8)**	1,4,5*	**0.965**	**0.978**	**0.955**	**0.972**	**0.907**	**0.948**	**0.914**	**0.939**	**0.838**	**0.910**	**0.866**	**0.914**	**0.877**
	1,3,4,5	0.013	0.007	0.021	0.010	0.043	0.019	0.039	0.025	0.077	0.041	0.063	0.040	0.054
	1,2,4,5	0.022	0.015	0.024	0.018	0.048	0.031	0.045	0.035	0.071	0.045	0.060	0.043	0.063
	1,2,3,4,5	0.000	0.000	0.000	0.000	0.002	0.002	0.002	0.001	0.014	0.004	0.011	0.003	0.006
**(2,9,0,4,8)**	1,4,5	0.013	0.022	0.005	0.007	0.002	0.012	0.001	0.003	0.000	0.000	0.000	0.003	0.000
	1,2,5	0.001	0.002	0.000	0.000	0.000	0.000	0.000	0.000	0.000	0.000	0.000	0.000	0.000
	1,3,4,5	0.001	0.003	0.001	0.001	0.004	0.005	0.001	0.003	0.004	0.005	0.001	0.003	0.001
	1,2,4,5*	**0.976**	**0.966**	**0.979**	**0.984**	**0.956**	**0.966**	**0.958**	**0.971**	**0.916**	**0.942**	**0.930**	**0.949**	**0.934**
	1,2,3,4,5	0.009	0.007	0.015	0.008	0.038	0.017	0.040	0.023	0.080	0.044	0.069	0.045	0.065
**(2,9,6,4,8)**	1,3,4,5	0.071	0.097	0.027	0.049	0.015	0.032	0.006	0.012	0.008	0.018	0.002	0.008	0.001
	1,2,4,5	0.010	0.020	0.006	0.009	0.000	0.003	0.000	0.000	0.001	0.003	0.000	0.001	0.000
	1,2,3,5	0.011	0.014	0.000	0.003	0.000	0.001	0.000	0.000	0.000	0.000	0.000	0.000	0.000
	1,2,3,4,5*	**0.908**	**0.869**	**0.967**	**0.939**	**0.985**	**0.964**	**0.994**	**0.988**	**0.991**	**0.979**	**0.998**	**0.991**	**0.999**

The (*) indicates the optimal model.

Significant values are in bold.

**Table 2:** Estimated selection probabilities of $\overline{\alpha }_{m,n}$, $\tilde{\alpha }_{m,n}$, ${\hat{\mathbf{\alpha }}}_{{{\mathbf{m,n}}}}$ and $\hat{\alpha }_{m,n}^{*}$ based on the least squares estimator and $\rho (x) = \min (x^{2} ,b^{2} )$.

True $$\beta ^{T}$$	Model	m = 15				m = 20				m = 25				$$\overset{\lower0.5em\hbox{$\smash{\scriptscriptstyle\smile}$}}{\alpha }_{40}$$
		$\overline{\alpha }_{15,40}$	${\hat{\mathbf{\alpha }}}_{{15{\mathbf{,}}40}}$	$\tilde{\alpha }_{15,40}$	$\hat{\alpha }_{15,40}^{*}$	$\overline{\alpha }_{20,40}$	${\hat{\mathbf{\alpha }}}_{{20{\mathbf{,}}40}}$	$\tilde{\alpha }_{20,40}$	$\hat{\alpha }_{20,40}^{*}$	$\overline{\alpha }_{25,40}$	${\hat{\mathbf{\alpha }}}_{{25{\mathbf{,}}40}}$	$\tilde{\alpha }_{25,40}$	$\hat{\alpha }_{25,40}^{*}$	
**(2,0,0,4,0)**	1,4*	**0.897**	**0.971**	**0.879**	**0.929**	**0.800**	**0.927**	**0.832**	**0.902**	**0.672**	**0.846**	**0.781**	**0.885**	**0.839**
	1,4,5	0.028	0.010	0.028	0.021	0.053	0.021	0.045	0.028	0.077	0.046	0.056	0.032	0.043
	1,3,4	0.029	0.010	0.029	0.018	0.071	0.018	0.057	0.027	0.116	0.054	0.076	0.037	0.047
	1,2,4	0.042	0.009	0.042	0.029	0.060	0.031	0.054	0.039	0.094	0.046	0.065	0.039	0.055
	1,3,4,5	0.001	0.000	0.001	0.001	0.006	0.001	0.004	0.001	0.013	0.003	0.007	0.002	0.004
	1,2,4,5	0.002	0.000	0.002	0.002	0.005	0.002	0.006	0.002	0.011	0.002	0.009	0.004	0.009
	1,2,3,4	0.001	0.000	0.001	0.000	0.005	0.000	0.002	0.001	0.012	0.003	0.006	0.001	0.003
	1,2,3,4,5	0.000	0.000	0.000	0.000	0.000	0.000	0.000	0.000	0.005	0.000	0.000	0.000	0.000
**(2,0,0,4,8)**	1,4,5*	**0.934**	**0.981**	**0.918**	**0.957**	**0.860**	**0.951**	**0.875**	**0.933**	**0.781**	**0.893**	**0.835**	**0.909**	**0.878**
	1,3,4,5	0.023	0.010	0.032	0.017	0.065	0.021	0.057	0.029	0.098	0.052	0.075	0.040	0.055
	1,2,4,5	0.043	0.009	0.050	0.026	0.068	0.028	0.061	0.038	0.100	0.049	0.077	0.050	0.061
	1,2,3,4,5	0.000	0.000	0.000	0.000	0.007	0.000	0.007	0.000	0.021	0.006	0.013	0.001	0.006
**(2,9,0,4,8)**	1,4,5	0.000	0.005	0.000	0.000	0.000	0.001	0.000	0.000	0.000	0.001	0.000	0.001	0.002
	1,3,4,5	0.000	0.000	0.000	0.001	0.000	0.000	0.001	0.001	0.000	0.001	0.001	0.001	0.001
	1,2,4,5*	**0.979**	**0.989**	**0.972**	**0982**	**0.932**	**0.972**	**0.933**	**0.968**	**0.875**	**0.939**	**0.910**	**0.951**	**0.933**
	1,2,3,4,5	0.021	0.006	0.027	0.017	0.068	0.027	0.066	0.031	0.125	0.059	0.089	0.047	0.064
**(2,9,6,4,8)**	1,3,4,5	0.008	0.036	0.001	0.007	0.002	0.010	0.001	0.005	0.000	0.005	0.001	0.001	0.001
	1,2,4,5	0.001	0.004	0.000	0.001	0.000	0.001	0.000	0.000	0.000	0.001	0.000	0.000	0.000
	1,2,3,5	0.000	0.001	0.000	0.000	0.000	0.000	0.000	0.000	0.000	0.000	0.000	0.000	0.000
	1,2,3,4,5*	**0.991**	**0.959**	**0.999**	**0.992**	**0.998**	**0.989**	**0.999**	**0.995**	**1.000**	**0.994**	**0.999**	**0.999**	**0.999**

The results are based on 1000 Monte Carlo simulations and K = 100 bootstrap replications.

Significant values are in bold.

The simulation resultspresented in Tables 1 and 2 are summarized as follows:

- The performance of the modified model selection procedure using the least squares estimator is comparable to the existing methods $$\overline{\alpha }_{m,n}$$, $$\tilde{\alpha }_{m,n}$$, $${\hat{\mathbf{\alpha }}}_{{{\mathbf{m,n}}}}$$ and the BIC($$\overset{\lower0.5em\hbox{$\smash{\scriptscriptstyle\smile}$}}{\alpha }_{n}$$).
- The proposed selection criterion outperforms the existent procedures in both cases, i.e., either using the squared loss function $\rho (x) = x^{2}$ or the robust loss function $\rho (x) = \min (x^{2} ,b^{2} )$.
- For the full model, if bootstrap sample size ***m*** increases, the estimated selection probabilities also increase. For example, in the case of ***m*** = 15, the correct percent is 93.9%, whereas, for ***m*** = 25, the correct percent is 99.1%.
- Moreover, model selection based on the robust loss function is superior to the squared loss function. For instance, in the case when the optimal model has all the predictors, then the modified model selection procedure $\hat{\alpha }_{15,40}^{*}$ using the squared loss function selects the optimal model 93.9% of the time, which is less than the 99.2% obtained by using a robust loss function.
- Furthermore, the modified selection criterion $\hat{\alpha }_{m,n}^{*}$ is less dependent on bootstrap sample size ***m*** as compared to the existent criteria $\overline{\alpha }_{m,n}$ and $\tilde{\alpha }_{m,n} .$

#### Simulated data and model selection consistency

To show model selection consistency and performance of the proposed criterion on simulated data, the following regression model with ***p*** = 5 is considered.

15 $$y_{i} = x_{i}^{T} \beta + \varepsilon_{i} ,\quad i = 1,2,...,n$$

where $\varepsilon_{i}$ is generated from standard normal distribution, the regression variables are generated from ${\rm N}(0,1),$ and added an intercept column of 1’s to produce the design matrix $X$. To generate the response variable $y_{i}$, we use Eq. (15).

The true data generating models are:

- $\beta_{1} = (1,0,0,1,0)$, i.e. the model had one nonzero variable,
- $\beta_{2} = (1,0,0,1,1)$ , i.e. the model had two nonzero variables,
- $\beta_{3} = (1,1,0,1,1)$, i.e. the model had three nonzero variables and

The estimated selection probabilities for $\hat{\alpha }_{m,n}^{*}$, $\tilde{\alpha }_{m,n}$, $\hat{\alpha }_{m,n}$ and $\overline{\alpha }_{m,n}$ are calculated for ***m*** = 24 and *n* = 40,80,120,160, based on ***L*** = 1000 simulations with bootstrap replications of ***K*** = 50 and are tabulated in Table 3.

**Table 3:** Selection probabilities of $\hat{\alpha }_{m,n}^{*}$ , $\tilde{\alpha }_{m,n}$,$\hat{\alpha }_{m,n}$ and $\overline{\alpha }_{m,n}$ based on LS-estimator and $\rho (x) = \min (x^{2} ,b^{2} )$.

True	Model	*m* = 24, *n* = 40				*m* = 24, *n* = 80				*m* = 24, *n* = 120				*m* = 24, *n* = 160			
		$\overline{\alpha }_{24,40}$	$\hat{\alpha }_{24,40}$	$\tilde{\alpha }_{24,40}$	$\hat{\alpha }_{24,40}^{*}$	$\overline{\alpha }_{24,80}$	$\hat{\alpha }_{24,80}$	$\tilde{\alpha }_{24,80}$	$\hat{\alpha }_{24,80}^{*}$	$\overline{\alpha }_{24,120}$	$\hat{\alpha }_{24,120}$	$\tilde{\alpha }_{24,120}$	$\hat{\alpha }_{24,120}^{*}$	$\overline{\alpha }_{24,160}$	$\hat{\alpha }_{24,160}$	$\tilde{\alpha }_{24,160}$	$\hat{\alpha }_{24,160}^{*}$
(1,0,0,1,0)	1	0.000	0.002	0.002	0.002	0.000	0.000	0.000	0.000	0.000	0.000	0.000	0.000	0.000	0.000	0.000	0.000
	1,4*	**0.405**	**0.731**	**0.672**	**0.785**	**0.752**	**0.886**	**0.851**	**0.897**	**0.893**	**0.953**	**0.923**	**0.948**	**0.953**	**0.978**	**0.954**	**0.969**
	1,4,5	0.158	0.088	0.104	0.071	0.065	0.034	0.039	0.030	0.038	0.016	0.031	0.017	0.012	0.007	0.012	0.010
	1,3,4	0.132	0.073	0.080	0.063	0.084	0.036	0.052	0.032	0.036	0.016	0.026	0.018	0.017	0.008	0.015	0.010
	1,2,4	0.172	0.087	0.102	0.069	0.071	0.040	0.046	0.036	0.029	0.014	0.018	0.015	0.016	0.007	0.019	0.011
	1,3,4,5	0.041	0.006	0.016	0.004	0.003	0.001	0.002	0.001	0.001	0.000	0.000	0.000	0.000	0.000	0.000	0.000
	1,2,4,5	0.048	0.008	0.014	0.004	0.015	0.002	0.009	0.003	0.002	0.000	0.001	0.001	0.001	0.000	0.000	0.000
	1,2,3,4	0.025	0.004	0.010	0.002	0.010	0.001	0.001	0.001	0.001	0.001	0.001	0.001	0.001	0.000	0.000	0.000
	1,2,3,4,5	0.019	0.001	0.000	0.000	0.000	0.000	0.000	0.000	0.000	0.000	0.000	0.000	0.000	0.000	0.000	0.000
(1,0,0,1,1)	1,5	0.000	0.001	0.000	0.001	0.000	0.000	0.000	0.000	0.000	0.000	0.000	0.000	0.000	0.000	0.000	0.000
	1,4	0.000	0.001	0.000	0.001	0.000	0.000	0.000	0.000	0.000	0.000	0.000	0.000	0.000	0.000	0.000	0.000
	1,4,5*	**0.566**	**0.821**	**0.784**	**0.865**	**0.828**	**0.939**	**0.896**	**0.938**	**0.930**	**0.971**	**0.950**	**0.969**	**0.975**	**0.990**	**0.968**	**0.984**
	1,3,4,5	0.175	0.080	0.092	0.064	0.079	0.025	0.049	0.026	0.035	0.014	0.027	0.016	0.011	0.007	0.016	0.007
	1,2,4,5	0.206	0.093	0.114	0.066	0.085	0.035	0.053	0.036	0.033	0.014	0.022	0.014	0.013	0.003	0.016	0.009
	1,2,3,4,5	0.053	0.004	0.010	0.003	0.008	0.001	0.002	0.000	0.002	0.001	0.001	0.001	0.001	0.000	0.000	0.000
(1,1,0,1,1)	1,2,4,5*	**0.779**	**0.921**	**0.894**	**0.938**	**0.922**	**0.971**	**0.953**	**0.973**	**0.965**	**0.983**	**0.975**	**0.982**	**0.991**	**0.994**	**0.987**	**0.991**
	1,2,3,4,5	0.221	0.079	0.106	0.062	0.078	0.029	0.047	0.027	0.035	0.017	0.025	0.018	0.009	0.006	0.013	0.009

The results are based on *L* = 1000 MC simulations and *K* = 50 bootstrap replications.

Significant values are in bold.

From the simulation results presented in Table 3, we see that our proposed criterion is comparatively consistent procedure for model selection in linear regression problems.

### Simulation setting 2

#### Simulated data from uniform distribution

In this subsection, the finite-sample performance of our modified criterion is compared with existing model selection procedures in the presence of outliers. The sample data is generated from the following model

$$y_{i} = 2 + 2x_{i1} + 0x_{i2} + \in_{i \, } \, i = 1,2,. . . ,64$$

where the design matrix X has columns generated as uniform on [− 1, 1]. The following six different error distributions are considered:

i. $\in_{1}$ is [3/8] outliers (i.e., [5/8]from a standard normal and [3/8] from a normal with $\mu = 30 - 2 - 2x_{1}$ and $\sigma = 1$);
ii. $\in_{2}$ is [1/4] outliers (i.e., [3/4] from a standard normal and [1/4] from a normal with $\mu = 30 - 2 - 2x_{1}$ and $\sigma = 1$);
iii. $\in_{3}$ is [1/8] outliers (i.e., [7/8] from a standard normal and [1/8] from a normal with $\mu = 30 - 2 - 2x_{1}$ and $\sigma = 1$);
iv. $\in_{4}$, the Gaussian distribution with μ = 0 and $\sigma = 1$;
v. $\in_{5}$, the Cauchy distribution;
vi. $\in_{6}$, the slash distribution(i.e. $\in_{6} \sim Z/U$ where $Z \sim N(0,1)\,\,\,$ and $U \sim U(0,1)$)

In Table 4, the following possible models are considered:

- Model (1) means, a model with intercept only;
- Model (1, 2) means a model having intercept and X_1_;
- Model (1, 3) means a model having intercept and X_2_;
- Model (1, 2, 3) means the full model.

**Table 4:** Estimated selection probabilities of $\overline{\alpha }_{24,64}$, $\hat{\alpha }_{24,64}$, $\tilde{\alpha }_{24,64}$ and $\hat{\alpha }_{24,64}^{*}$ based on MM-estimator and LS-estimator.

Errors	Model	MM-estimator								LS-estimator			
		Simple bootstrap				Stratified bootstrap				Simple bootstrap			
		$\overline{\alpha }_{24,64}$	$\hat{\alpha }_{24,64}$	$\tilde{\alpha }_{24,64}$	$\hat{\alpha }_{24,64}^{*}$	$\overline{\alpha }^{S8}_{24,64}$	$\hat{\alpha }^{S8}_{24,64}$	$\tilde{\alpha }^{S8}_{24,64}$	$\hat{\alpha }^{*S8}_{24,64}$	$\overline{\alpha }_{24,64}$	$\hat{\alpha }_{24,64}$	$\tilde{\alpha }_{24,64}$	$\hat{\alpha }_{24,64}^{*}$
$\in_{1}$	1	0.368	0.392	0.267	0.257	0.000	0.000	0.001	0.001	0.756	1.000	1.000	1.000
	1,3	0.000	0.000	0.000	0.000	0.000	0.000	0.000	0.000	0.244	0.000	0.000	0.000
	1,2*	**0.290**	**0.362**	**0.722**	**0.738**	**0.916**	**0.968**	**0.997**	**0.997**	**0.000**	**0.000**	**0.000**	**0.000**
	1,2,3	0.342	0.246	0.011	0.005	0.084	0.032	0.002	0.002	0.000	0.000	0.000	0.000
$\in_{2}$	1	0.000	0.000	0.000	0.000	0.000	0.000	0.000	0.000	0.999	1.000	1.000	1.000
	1,3	0.000	0.000	0.000	0.000	0.000	0.000	0.000	0.000	0.000	0.000	0.000	0.000
	1,2*	**0.935**	**0.968**	**0.983**	**0.986**	**0.886**	**0.958**	**0.978**	**0.984**	**0.001**	**0.000**	**0.000**	**0.000**
	1,2,3	0.065	0.032	0.017	0.014	0.0114	0.042	0.022	0.016	0.000	0.000	0.000	0.000
$\in_{3}$	1	0.000	0.000	0.000	0.000	0.000	0.000	0.000	0.000	1.000	1.000	1.000	1.000
	1,3	0.000	0.000	0.000	0.000	0.000	0.000	0.000	0.000	0.000	0.000	0.000	0.000
	1,2*	**0.894**	**0.952**	**0.947**	**0.963**	**0.869**	**0.946**	**0.943**	**0.960**	**0.000**	**0.000**	**0.000**	**0.000**
	1,2,3	0.106	0.048	0.053	0.037	0.131	0.054	0.057	0.040	0.000	0.000	0.000	0.000
$\in_{4}$	1	0.000	0.000	0.000	0.000	0.000	0.000	0.000	0.000	0.000	0.000	0.000	0.000
	1,3	0.000	0.000	0.000	0.000	0.000	0.000	0.000	0.000	0.000	0.000	0.000	0.000
	1,2*	**0.891**	**0.961**	**0.933**	**0.956**	**0.859**	**0.939**	**0.919**	**0.948**	**0.869**	**0.949**	**0.929**	**0.958**
	1,2,3	0.109	0.039	0.067	0.044	0.141	0.061	0.081	0.052	0.131	0.051	0.071	0.042
$\in_{5}$	1	0.008	0.016	0.012	0.018	0.005	0.013	0.010	0.013	0.770	0.823	0.841	0.866
	1,3	0.000	0.000	0.000	0.000	0.001	0.000	0.000	0.000	0.001	0.001	0.000	0.000
	1,2*	**0.954**	**0.974**	**0.961**	**0.973**	**0.929**	**0.966**	**0.952**	**0.969**	**0.227**	**0.175**	**0.159**	**0.134**
	1,2,3	0.038	0.010	0.027	0.009	0.065	0.021	0.038	0.018	0.002	0.001	0.000	0.000
$\in_{6}$	1	0.062	0.139	0.081	0.124	0.042	0.093	0.067	0.099	0.828	0.882	0.909	0.921
	1,3	0.004	0.005	0.004	0.003	0.008	0.005	0.004	0.004	0.005	0.002	0.001	0.001
	1,2*	**0.885**	**0.842**	**0.882**	**0.855**	**0.879**	**0.878**	**0.892**	**0.875**	**0.164**	**0.116**	**0.090**	**0.078**
	1,2,3	0.049	0.014	0.033	0.018	0.071	0.024	0.037	0.022	0.003	0.000	0.000	0.000

The outputs are based on 1000 MCsimulations and K = 100 bootstrap replications. The $\rho (x) = \min (x^{2} ,2^{2} )$ is used for all selection criteria.

Significant values are in bold.

Following^19^, the MM-estimator of^20^ is used to fit the robust regression models. For this purpose, the rlm ( ) function in **R** is used for estimating the regression parameters. Furthermore, the LS estimates are computed for comparison with MM-estimates. As mentioned by^28^, when the proportion of extreme observations in some of the bootstrap samples is higher than in the original sample, then the bootstrap distribution may provide a very poor estimator of the distribution of the MM-estimates. To deal with this numerical instability, we use the stratified bootstrap with equal-sized strata. In this approach, bootstrap samples are constructed so that the distribution of the residuals in each bootstrap sample reflects the one observed in the original data set. The selection probabilities based on ***L*** = 1000 simulations with bootstrap replications of **K** = 100 are given in Table 4.

From the simulation results presented in Table 4, it is clear that the modified selection procedure using the robust $\rho (.)$ function and MM-estimator is robust in the presence of highly contaminated data. For example, the percent correct is 73.8% for un-stratified bootstrap, whereas the percent correct is 99.7% for stratified bootstrap under the contaminated normal situation $\in_{1}$. For all error distributions, the modified robust criterion outperforms the existence criteria. The simulation studies suggest that when errors are non-normal, then using robust regression is superior to using LS, but in the case of normal errors, robust regression is inferior to LS. Furthermore, in the presence of outliers and heavy-tailed error distributions, the modified robust criterion using MM-estimator outperforms LS-estimator by a large margin. For example, under $\in_{5}$ error distribution, for MM-estimator the percent correct is 96.9%, whereas, the percent correct is 13.4% for LS-estimator. These results demonstrate that the modified robust procedure has good robustness characteristics with contaminated normal and heavy-tailed distributions, whereas the LS procedure performs very poorly in both cases. This clearly proves the lack of robustness of the LS procedure in the presence of outliers and heavy-tailed distributions. An excellent amount of improvement is obtained in the bootstrap model selection procedure by using the combined criterion as observed in the above simulation study.

#### Modified solid waste data of Gunst and Mason

To evaluate the performance of our proposed robust model selection method, we modified the Gunst and Mason data by planting 10% and 20% outliers. The response vector is generated as

$${\mathbf{Y}}_{{\mathbf{i}}} {\mathbf{ = \beta }}_{{\mathbf{0}}} {\mathbf{X}}_{{{\mathbf{i0}}}} {\mathbf{ + \beta }}_{{\mathbf{1}}} {\mathbf{X}}_{{{\mathbf{i1}}}} {\mathbf{ + \beta }}_{{\mathbf{2}}} {\mathbf{X}}_{{{\mathbf{i2}}}} {\mathbf{ + \beta }}_{{\mathbf{3}}} {\mathbf{X}}_{{{\mathbf{i3}}}} {\mathbf{ + \beta }}_{{\mathbf{4}}} {\mathbf{X}}_{{{\mathbf{i4}}}} + \varepsilon_{i} ,\quad i = 1,2,...,40$$

where $X_{0}$ is the column of 1’s; and the values of $X_{1} ,X_{2} ,X_{3}$ and $X_{4}$ are taken from the solid waste data of^26^. To create high-leverage points, we replace the first four to eight observations of each regressor variable value by 20. The true generating model has two non-zero predictors, i.e. ${{\varvec{\upbeta}}}^{{\mathbf{T}}} {\mathbf{ = (2,0,0,4,8)}}$ and we choose the following five different error distributions to represent various deviations from normality:

i. $\in_{1}$ is 10% wild (i.e., 90% from a standard normal and 10% from a normal with, $\mu = 0\;{\text{ and }}\;\sigma = 0.7$);
ii. $\in_{2}$ is 20% wild (i.e., 80% from a standard normal and 20% from a normal with ,$\mu = 0 \, \;{\text{and}}\; \, \sigma = 5$) ;
iii. $\in_{3}$.is t(3) (i.e., t-distribution with 3 degrees of freedom);
iv. $\in_{4}$, is standard normal;
v. $\in_{5}$, is Cauchy distribution with location = 0 and scale = 1.

The selection probabilities of $\overline{\alpha }_{m,n}$, $\hat{\alpha }_{m,n}$, $\tilde{\alpha }_{m,n}$ and $\hat{\alpha }_{m,n}^{*}$ on the basis of stratified bootstrap with the MM estimator are computed. The selection probabilities based on ***L*** = 1000 simulations with bootstrap replications of **K** = 50 are given in Table 5.

**Table 5:** Estimated selection probabilities of $\overline{\alpha }_{m,n}$, $\tilde{\alpha }_{m,n}$, ${\hat{\mathbf{\alpha }}}_{{{\mathbf{m,n}}}}$ and $\hat{\alpha }_{m,n}^{*}$ based on MM estimator.

Errors	True ${\varvec{\beta}}^{\rm T}$	Model	10% X-outliers				20% X-outliers			
			$\overline{\alpha }_{16,40}$	${\hat{\mathbf{\alpha }}}_{{16{\mathbf{,}}40}}$	$\tilde{\alpha }_{16,40}$	$\hat{\alpha }_{16,40}^{*}$	$\overline{\alpha }_{16,40}$	${\hat{\mathbf{\alpha }}}_{{16{\mathbf{,}}40}}$	$\tilde{\alpha }_{16,40}$	$\hat{\alpha }_{16,40}^{*}$
$\in_{1}$	**(2,0,0,4,8)**	1,5	0.002	0.004	0.002	0.002	0.008	0.034	0.007	0.018
		1,4,5*	**0.912**	**0.966**	**0.902**	**0.940**	**0.823**	**0.883**	**0.824**	**0.876**
		1,3,5	0.005	0.006	0.008	0.008	0.034	0.035	0.036	0.033
		1,2,5	0.002	0.004	0.003	0.003	0.011	0.016	0.013	0.015
		1,3,4,5	0.036	0.008	0.034	0.019	0.051	0.018	0.052	0.027
		1,2,4,5	0.041	0.012	0.045	0.026	0.062	0.014	0.048	0.026
		1,2,3,4,5	0.002	0.000	0.006	0.002	0.011	0.000	0.020	0.005
$\in_{2}$	**(2,0,0,4,8)**	1,5	0.008	0.032	0.017	0.027	0.018	0.093	0.029	0.056
		1,4,5*	**0.606**	**0.793**	**0.684**	**0.781**	**0.531**	**0.714**	**0.642**	**0.717**
		1,3,5	0.021	0.030	0.033	0.033	0.034	0.039	0.038	0.046
		1,2,5	0.013	0.028	0.016	0.021	0.019	0.017	0.021	0.022
		1,3,4,5	0.091	0.027	0.072	0.041	0.110	0.039	0.085	0.046
		1,2,4,5	0.255	0.089	0.170	0.94	0.282	0.098	0.179	0.109
		1,2,3,4,5	0.006	0.001	0.008	0.003	0.004	0.000	0.001	0.001
$\in_{3}$	**(2,0,0,4,8)**	1,5	0.004	0.021	0.003	0.007	0.026	0.077	0.027	0.055
		1,4	0.000	0.000	0.000	0.000	0.000	0.001	0.001	0.001
		1,4,5*	**0.887**	**0.929**	**0.886**	**0.920**	**0.807**	**0.815**	**0.817**	**0.824**
		1,3,5	0.015	0.022	0.019	0.021	0.052	0.053	0.057	0.052
		1,2,5	0.010	0.012	0.013	0.017	0.032	0.028	0.031	0.030
		1,3,4,5	0.045	0.012	0.042	0.018	0.042	0.013	0.030	0.017
		1,2,4,5	0.036	0.0.03	0.032	0.014	0.039	0.012	0.034	0.018
		1,2,3,4,5	0.003	0.001	0.005	0.003	0.002	0.001	0.003	0.003
$\in_{4}$	**(2,0,0,4,8)**	1,5	0.001	0.004	0.001	0.002	0.005	0.019	0.008	0.015
		1,4,5*	**0.921**	**0.965**	**0.913**	**0.948**	**0.863**	**0.904**	**0.859**	**0.897**
		1,3,5	0.005	0.007	0.003	0.005	0.029	0.035	0.032	0.032
		1,2,5	0.003	0.005	0.005	0.004	0.012	0.012	0.009	0.010
		1,3,4,5	0.039	0.011	0.034	0.021	0.046	0.017	0.040	0.022
		1,2,4,5	0.029	0.008	0.039	0.019	0.038	0.011	0.039	0.020
		1,2,3,4,5	0.002	0.000	0.005	0.000	0.007	0.001	0.013	0.003
$\in_{5}$	**(2,0,0,4,8)**	1,5	0.045	0114	0.050	0.081	0.162	0.274	0.162	0.251
		1.4	0.001	0.002	0.001	0.001	0.007	0.018	0.009	0.011
		1,4,5*	**0.839**	**0.820**	**0.842**	**0.843**	**0.670**	**0.611**	**0.692**	**0.637**
		1,3,5	0.046	0.034	0.044	0.035	0.055	0.041	0.056	0.046
		1,2,5	0.020	0.022	0.019	0.022	0.045	0.042	0.037	0.033
		1,3,4,5	0.026	0.004	0.017	0.009	0.024	0.009	0.016	0.008
		1,2,4,5	0.018	0.003	0.020	0.008	0.0028	0.005	0.019	0.013
		1,2,3,5	0.002	0.000	0.003	0.000	0.007	0.000	0.007	0.000
		1,2,3,4,5	0.003	0.001	0.002	0.001	0.002	0.000	0.002	0.001

The results are based on 1000 Monte Carlo simulations and K = 50 bootstrap replications.

Significant values are in bold.

Table 5 demonstrates the simulation results with 10% and 20% of outliers in the covariates and five different error distributions as discussed in the simulation setting. If we look at the results, we see that the performance of our robust procedure is very good for $\in_{4}$ amongst all error distributions while it does not perform very well for $\in_{5}$ in the presence of x-outliers. The selection probabilities for error distribution $\in_{3}$ are similar to that of $\in_{4}$. Furthermore, the selection probabilities are good for distribution $\in_{1}$ (10% symmetric wild case) as compared to contamination type $\in_{2}$ (20% symmetric wild case). Overall, the selection probabilities for each of the criteria decrease when the percentage of both x- and y-outliers goes up. Moreover, selection probabilities in the presence of response outliers and covariate outliers, the performance of our proposed model selection criterion based on MM-estimation is comparable to the existing criteria even when the contamination level changes from i.e., 10% to 20%.

## Data example (Stack loss data)

In this section, we analyze the Stack loss data presented by^29^. This dataset consists of three explanatory variables, and it contains four outliers,namely observations 1, 3, 4, and 21.The response is the Stack loss (y) observed on ***n*** = 21 observations. The explanatory variables are theFlow of cooling air (X_1_), Cooling Water Temperature (X_2_), and Concentration of acid (X_3_).We applied our robust method $\hat{\alpha }_{m,n}^{*}$, the existing methods, and the traditional methods on the data. Table 5 presents a summary of selected best models.

Table 6 shows the classical methodsselect the full model, whereas robust criteria agreed with the importance of the two variables, X_1_ and X_2_.The best model according to our criterion includes X_1_ and X_2_.

**Table 6:** Selected best model for the stack loss data using a range of model selection procedures.

Selected variables	$\overline{\alpha }_{10,21}$	$\hat{\alpha }_{10,21}$	$\tilde{\alpha }_{10,21}$	$\hat{\alpha }_{10,21}^{*}$	AIC	BIC
X_1_	2.97	3.50	4.42	4.82	4.61	4.71
X_2_	3.02	3.59	4.58	5.00	2.19	2.28
X_3_	3.48	4.20	5.32	5.86	3.21	3.31
X_1_, X_2_	**1.70**	**2.55**	**3.23**	**3.87**	1.55	1.70
X_1_, X_3_	2.38	3.42	4.67	5.45	2.69	2.84
X_2_, X_3_	2.09	3.49	3.72	4.77	1.47	1.62
X_1_, X_2_, X_3_	1.81	3.05	3.90	5.28	**1.34**	**1.54**

Significant values are in bold.

## Conclusion

In this article, we have presented a novel procedure for robust model selection in linear regression. The criterion is a modification to the bootstrap model selection method based on robust estimator proposed by^19^. The simulation results reveal that the performance of model selection is improved when using the OOB error in the present studies. Moreover, the undue effect of outliers is controlled by using both a robust MM-estimator and a bounded loss function in the proposed criterion. The proposed model selection criterion can maintain their robust properties in the presence of response outliers and covariate outliers. The proposed criterion is compared with other robust model selection criteria described in previous literature.

We observed that in the presence of outliers and heavy-tailed error distributions, the MM-estimator outperformed the least squares estimator by a large margin. This clearly proved the lack of robustness of the least squares procedure in the presence of outliers and in heavy-tailed distributions. Furthermore, when errors are non-normal, then robust regression is found superior to least squares, but in the case of normal errors, robust regression is found inferior to least squares.

From simulation-based and real-data based results, we conclude that our modified robust model selection procedure is consistent and works well in situations where outliers are present in the data. As observed in our simulation study, an excellent amount of improvement is gained by minimizing the combined criterion, rather than minimizing the penalized loss function or the modified conditional expected prediction loss separately. Furthermore, our robust model selection criterion will perform better when the data generating model is small.

## Supplementary Information

Supplementary Information.

## References

[1] Akaike H (1970). Statistical predictor identification. Ann. Inst. Stat. Math..

[2] Mallows CL (1973). Some comments on C_p_. Technometrics.

[3] Schwarz G (1978). Estimating the dimension of a model. Ann. Stat..

[4] Breiman L (1995). Better subset regression using the nonnegative garrote. Technometrics.

[5] Shao J (1996). Bootstrap model selection. J. Am. Stat. Assoc..

[6] Rabbi F, Khan S, Khalil A, Mashwani WK, Shafiq M, Gӧktas P, Unvan YA (2021). Model selection in linear regression using paired bootstrap. Commun. Stat. Theory Methods.

[7] Ronchetti E (1985). Robust model selection in regression. Stat. Probab. Lett..

[8] Ronchetti E, Staudte RG (1994). A robust version of Mallows's C_p_. J. Am. Stat. Assoc..

[9] Sommer S, Staudte RG (1995). Robust variable selection in regression in the presence of outliers and leverage points. Aust. J. Stat..

[10] Sommer S, Huggins RM (1996). Variables selection using the Wald test and a robust C_P_. Appl. Stat..

[11] Ronchetti E, Field C, Blanchard W (1997). Robust linear model selection by cross-validation. J. Am. Stat. Assoc..

[12] Wisnowski JW, Simpson JR, Montgomery DC, Runger GC (2003). Resampling methods for variable selection in robust regression. Comput. Stat. Data Anal..

[13] Salibian-Barrera M, Van Aelst S (2008). Robust model selection using fast and robust bootstrap. Comput. Stat. Data Anal..

[14] Müller S, Welsh A (2009). Robust model selection in generalized linear models. Stat. Sin..

[15] Tharmaratnam K, Claeskens G (2013). A comparison of robust versions of the AIC based on M-, S-and MM-estimators. Statistics.

[16] Saleh S (2014). Robust AIC with high breakdown scale estimate. J. Appl. Math..

[17] Sakate D, Kashid D (2016). A new robust model selection method in GLM with application to ecological data. Environ. Syst. Res..

[18] Rabbi F, Khan S, Khalil A, Salahuddin N (2019). Robust linear model selection using paired bootstrap. Indian J. Sci. Technol..

[19] Müller S, Welsh A (2005). Outlier robust model selection in linear regression. J. Am. Stat. Assoc..

[20] Yohai VJ (1987). High breakdown-point and high-efficiency robust estimates for regression. Ann. Stat..

[21] Breiman L (2001). Random forests. Mach. Learn..

[22] Zhang G-Y, Zhang C-X, Zhang J-S (2010). Out-of-bag estimation of the optimal hyperparameter in subbag ensemble method. Commun. Stat. Simul. Comput..

[23] Imon AHMR, Ali MM (2005). Bootstrapping regression residuals. J. Korean Data Inf. Sci. Soc..

[24] Cochran WG (1977). Sampling Technique.

[25] Hampel, F. Some aspects of model choice in robust statistics. In *Proceedings of the 44th Session of the ISI, Madrid, Book*, Vol. 2 767–771 (1983).

[26] Ronchetti E (1997). Robustness aspects of model choice. Stat. Sin..

[27] Gunst RF, Mason RL (1980). Regression Analysis and its Applications.

[28] Salibian-Barrera M, Zamar RH (2002). Bootstrapping robust estimates of regression. Ann. Stat..

[29] Brownlee KA (1965). Statistical Theory and Methodology in Science and Engineering.

