# Supplementary material for: Robust model selection using the out-of-bag bootstrap in linear regression
Source: Sci Rep. 2022 Jun 29;12:10992. doi: 10.1038/s41598-022-14398-1 (PMC9243146; doi:10.1038/s41598-022-14398-1)
Supplement: Supplementary file 1 — Supplementary Information. [file 41598_2022_14398_MOESM1_ESM.docx]

# Appendix A: R- Code

## A-1. R-code for the robust version of Shao's (1996) Criterion

x1=matrix(runif(n,-1,1))

x2=matrix(runif(n,-1,1))

X=cbind(1L,x1,x2)

ASM = function(k.s,intcpt = TRUE){

if (intcpt == FALSE){p = p+1}

A = NULL

for(i in 1:p){

A0 = cbind(0L,A)

A1 = cbind(1L,A)

A = rbind(A0,A1)

}# end i-for-loop

if (intcpt == TRUE){

A = cbind(1L,A)

}

return(A[order(apply(A,1,sum)),])

} # end ASM function

A=ASM(p)

p.alpha=apply(A,1,sum)

n.a = dim(A)[1]

m.1=NULL

M = matrix(0,n.a,1)

M.2 = matrix(0,n.a,1)

for(l in 1:L){

set.seed(l)

e=rnorm(n,0,1)

y=X%*%beta+e

y=X%*%beta+e

e1=rnorm(n,30-2-2*x1,1)

y[1:16]=X%*%beta+e1

n1=0

M2=matrix(0,n.a,1)

Mn=matrix(0,n.a,1)

dat=data.frame(y,X)

Mf.MM = rlm(y~-1+X,data=dat,method="MM",maxit=200)

scale = mad(Mf.MM$res)

r=Mf.MM$res

rank=rank(abs(r))

s1=which(rank<=n/8)

s2=which(rank>n/8 & rank<=2*n/8)

s3=which(rank>2*n/8 & rank<=3*n/8)

s4=which(rank>3*n/8 & rank<=4*n/8)

s5=which(rank>4*n/8 & rank<=5*n/8)

s6=which(rank>5*n/8 & rank<=6*n/8)

s7=which(rank>6*n/8 & rank<=7*n/8)

s8=which(rank>7*n/8 & rank<=n)

for(k in 1:K){

bs.length = 0

while (bs.length < p+1){

bs=c(sample(s1,m/8,replace=TRUE),sample(s2,m/8,replace=TRUE),sample(s3,m/8,replace=TRUE),sample(s4,m/8,replace=TRUE),sample(s5,m/8,replace=TRUE),sample(s6,m/8,replace=TRUE),sample(s7,m/8,replace=TRUE),sample(s8,m/8,replace=TRUE))

bs.length=dim(table(bs))

}

for(a in 1:n.a){

X1=as.matrix(X[,A[a,]>0])

lm.fit.a = rlm(y[bs]~ -1 + X1[bs,],method="MM",maxit=200)

R.b =y-X1%*%as.matrix(coef(lm.fit.a),ncol=1)

M2[a]=M2[a]+sum(apply(cbind(R.b^2/(scale^2),b^2),1,min))

}#closing iterating over all models

n1=n1+length(R.b)

}

for(a in 1:n.a){

X1=as.matrix(X[,A[a,]>0])

lm.fit.a = lm(y~ -1 + X1)

Mn[a]=scale^2*(M2[a]/n1)

}

M[which.min(Mn)]=M[which.min(Mn)]+1

} #closing the L loops to be run

m.1=cbind(m.1,M)

## A-2. R-code for Rabbi et al. (2019 ) Criterion

x1=matrix(runif(n,-1,1))

x2=matrix(runif(n,-1,1))

X=cbind(1L,x1,x2)

ASM = function(k.s,intcpt = TRUE){

if (intcpt == FALSE){p = p+1}

A = NULL

for(i in 1:p){

A0 = cbind(0L,A)

A1 = cbind(1L,A)

A = rbind(A0,A1)

}# end i-for-loop

if (intcpt == TRUE){

A = cbind(1L,A)

}

return(A[order(apply(A,1,sum)),])

} # end ASM function

A=ASM(p)

p.alpha=apply(A,1,sum)

n.a = dim(A)[1]

m.1=NULL

M = matrix(0,n.a,1)

M.2 = matrix(0,n.a,1)

for(l in 1:L){

set.seed(l)

e=rnorm(n,0,1)

y=X%*%beta+e

e1=rnorm(n,30-2-2*x1,1)

y[1:16]=X%*%beta+e1

n1=0

M2=matrix(0,n.a,1)

Mn=matrix(0,n.a,1)

dat=data.frame(y,X)

Mf.MM = rlm(y~-1+X,data=dat,method="MM",maxit=200)

scale = mad(Mf.MM$res)

r=Mf.MM$res

rank=rank(abs(r))

s1=which(rank<=n/8)

s2=which(rank>n/8 & rank<=2*n/8)

s3=which(rank>2*n/8 & rank<=3*n/8)

s4=which(rank>3*n/8 & rank<=4*n/8)

s5=which(rank>4*n/8 & rank<=5*n/8)

s6=which(rank>5*n/8 & rank<=6*n/8)

s7=which(rank>6*n/8 & rank<=7*n/8)

s8=which(rank>7*n/8 & rank<=n)

for(k in 1:K){

bs.length = 0

while (bs.length < p+1){

bs=c(sample(s1,m/8,replace=TRUE),sample(s2,m/8,replace=TRUE),sample(s3,m/8,replace=TRUE),sample(s4,m/8,replace=TRUE),sample(s5,m/8,replace=TRUE),sample(s6,m/8,replace=TRUE),sample(s7,m/8,replace=TRUE),sample(s8,m/8,replace=TRUE))

y.st=y[bs]

bs.length=dim(table(bs))

}

for(a in 1:n.a){

X1=as.matrix(X[,A[a,]>0])

X.st=as.matrix(X1[bs,])

lm.fit.a = rlm(y.st~ -1 + X.st,method="MM",maxit=200)

R.b =y[-bs]-as.matrix(X1[-bs,])%*%as.matrix(coef(lm.fit.a),ncol=1)

M2[a]=M2[a]+sum(apply(cbind(R.b^2/(scale^2),b^2),1,min))

}#closing iterating over all models

n1=n1+length(R.b)

} #closing iterating over the bootstrap replications

for(a in 1:n.a){

X1=as.matrix(X[,A[a,]>0])

lm.fit.a = rlm(y~ -1 + X1,method="MM",maxit=100)

Mn[a]=scale^2*(M2[a]/n1)

} #closing the n.a models to be tested

M[which.min(Mn)]=M[which.min(Mn)]+1

print(l)

} #closing the L loops to be run

m.1=cbind(m.1,M)

## A-3. R-code for Müller and Welsh (2005) Criterion

x1=matrix(runif(n,-1,1))

x2=matrix(runif(n,-1,1))

X=cbind(1L,x1,x2)

ASM = function(k.s,intcpt = TRUE){

if (intcpt == FALSE){p = p+1}

A = NULL

for(i in 1:p){

A0 = cbind(0L,A)

A1 = cbind(1L,A)

A = rbind(A0,A1)

}

if (intcpt == TRUE){

A = cbind(1L,A)

}

return(A[order(apply(A,1,sum)),])

} # end ASM function

A=ASM(p)

p.alpha=apply(A,1,sum)

n.a = dim(A)[1]

m.1=NULL

M = matrix(0,n.a,1)

M.1 = matrix(0,n.a,1)

M.2 = matrix(0,n.a,1)

for(l in 1:L){

set.seed(l)

e=rnorm(n,0,1)

y=X%*%beta+e

e1=rnorm(n,30-2-2*x1,1)

y[1:16]=X%*%beta+e1

n1=0

M1=matrix(0,n.a,1)

M2=matrix(0,n.a,1)

Mn=matrix(0,n.a,1)

dat=data.frame(y,X)

Mf.MM = rlm(y~-1+X,data=dat,method="MM",maxit=200)

scale = mad(Mf.MM$res)

r=Mf.MM$res

rank=rank(abs(r))

s1=which(rank<=n/8)

s2=which(rank>n/8 & rank<=2*n/8)

s3=which(rank>2*n/8 & rank<=3*n/8)

s4=which(rank>3*n/8 & rank<=4*n/8)

s5=which(rank>4*n/8 & rank<=5*n/8)

s6=which(rank>5*n/8 & rank<=6*n/8)

s7=which(rank>6*n/8 & rank<=7*n/8)

s8=which(rank>7*n/8 & rank<=n)

for(k in 1:K){

bs.length = 0

while (bs.length < p+1){

bs=c(sample(s1,m/8,replace=TRUE),sample(s2,m/8,replace=TRUE),sample(s3,m/8,replace=TRUE),sample(s4,m/8,replace=TRUE),sample(s5,m/8,replace=TRUE),sample(s6,m/8,replace=TRUE),sample(s7,m/8,replace=TRUE),sample(s8,m/8,replace=TRUE))

y.st=y[bs]

bs.length=dim(table(bs))

}

for(a in 1:n.a){

X1=as.matrix(X[,A[a,]>0])

lm.fit.a = rlm(y[bs]~ -1 + X1[bs,],method="MM",maxit=200)

R.b =y-X1%*%as.matrix(coef(lm.fit.a),ncol=1)

M2[a]=M2[a]+sum(apply(cbind(R.b^2/(scale^2),b^2),1,min))

}#closing iterating over all models

n1=n1+length(R.b)

} #closing iterating over the bootstrap replications

for(a in 1:n.a){

X1=as.matrix(X[,A[a,]>0])

lm.fit.a = rlm(y~ -1 + X1,method="MM",maxit=100)

M1[a] = 1/n*(sum(apply(cbind(lm.fit.a$res^2/(scale^2),b^2),1,min)) +log(n)*p.alpha[a])

Mn[a]=scale^2*(M1[a]+M2[a]/n1)

} #closing the n.a models to be tested

M[which.min(Mn)]=M[which.min(Mn)]+1

M.2[which.min(M2)]=M.2[which.min(M2)]+1

M.1[which.min(M1)]=M.1[which.min(M1)]+1

print(l)

} #closing the L loops to be run

m.1=cbind(m.1,M,M.1,M.2)

##

## A-4. R-code for our proposed criterion

x1=matrix(runif(n,-1,1))

x2=matrix(runif(n,-1,1))

X=cbind(1L,x1,x2)

ASM = function(k.s,intcpt = TRUE){

if (intcpt == FALSE){p = p+1}

A = NULL

for(i in 1:p){

A0 = cbind(0L,A)

A1 = cbind(1L,A)

A = rbind(A0,A1)

}

if (intcpt == TRUE){

A = cbind(1L,A)

}

return(A[order(apply(A,1,sum)),])

} # end ASM function

A=ASM(p)

p.alpha=apply(A,1,sum)

n.a = dim(A)[1]

m.1=NULL

M = matrix(0,n.a,1)

M.1 = matrix(0,n.a,1)

M.2 = matrix(0,n.a,1)

for(l in 1:L){

e=rnorm(n,0,1)

y=X%*%beta+e

e1=rnorm(n,30-2-2*x1,1)

y[1:16]=X%*%beta+e1

n1=0

M1=matrix(0,n.a,1)

M2=matrix(0,n.a,1)

Mn=matrix(0,n.a,1)

dat=data.frame(y,X)

Mf.MM = rlm(y~-1+X,data=dat,method="MM",maxit=200)

scale = mad(Mf.MM$res)

r=Mf.MM$res

rank=rank(abs(r))

s1=which(rank<=n/8)

s2=which(rank>n/8 & rank<=2*n/8)

s3=which(rank>2*n/8 & rank<=3*n/8)

s4=which(rank>3*n/8 & rank<=4*n/8)

s5=which(rank>4*n/8 & rank<=5*n/8)

s6=which(rank>5*n/8 & rank<=6*n/8)

s7=which(rank>6*n/8 & rank<=7*n/8)

s8=which(rank>7*n/8 & rank<=n)

for(k in 1:K){

bs.length = 0

while (bs.length < p+1){

bs = c(sample(s1,m/8,replace=TRUE),sample(s2,m/8,replace=TRUE),sample(s3,m/8,replace=TRUE), sample(s4,m/8,replace=TRUE),sample(s5,m/8,replace=TRUE),sample(s6,m/8,replace=TRUE),sample(s7,m/8,replace=TRUE),sample(s8,m/8,replace=TRUE))

y.st=y[bs]

bs.length=dim(table(bs))

}

for(a in 1:n.a){

X1=as.matrix(X[,A[a,]>0])

X.st=as.matrix(X1[bs,])

lm.fit.a = rlm(y.st~ -1 + X.st,method="MM",maxit=200)

R.b =y[-bs]-as.matrix(X1[-bs,])%*%as.matrix(coef(lm.fit.a),ncol=1)

M2[a]=M2[a]+sum(apply(cbind(R.b^2/(scale^2),b^2),1,min))

} #closing iterating over all models

nK.corrected = nK.corrected+length(R.b)

} #closing iterating over the bootstrap replications

for(a in 1:n.a){

X1=as.matrix(X[,A[a,]>0])

lm.fit.a = rlm(y~ -1 + X1,method="MM",maxit=100)

M1[a] = 1/n*(sum(apply(cbind(lm.fit.a$res^2/(scale^2),b^2),1,min))+log(n)*p.alpha[a])

Mn[a]=scale^2*(M1[a]+M2[a]/nK.corrected)

} #closing the n.a models to be tested

M[which.min(Mn)]=M[which.min(Mn)]+1

M.2[which.min(M2)]=M.2[which.min(M2)]+1

M.1[which.min(M1)]=M.1[which.min(M1)]+1

} #closing the L loops to be run

m.1=cbind(m.1,M,M.1,M.2)

## A-5. R-code for our proposed criterion using modified solid waste data of Gunst and Mason

X.full=matrix(rnorm(n*p),ncol=p)

X.full[,1] = c(0.36, 1.32, 0.06, 0.16, 0.01, 0.02, 0.56, 0.98,0.32, 0.01, 0.15, 0.24, 0.11, 0.08, 0.61, 0.03,0.06, 0.02, 0.04, 0.00, 0.09, 0.02, 0.02, 0.05,0.11, 0.18, 0.04, 0.85, 0.17, 0.08, 0.38, 0.11,0.39, 0.43, 0.57, 0.13, 0.04, 0.13, 0.20, 0.07);

X.full[,2] =c(0.53, 2.52, 0.09, 0.41, 0.02, 0.07, 0.62, 1.06,

0.20, 0.00, 0.25, 0.28, 0.35, 0.13, 0.85, 0.03,

0.11, 0.08, 0.24, 0.02, 0.18, 0.16, 0.11, 0.24,

0.39, 0.11, 0.09, 1.33, 0.32, 0.12, 0.18, 0.13,

0.38, 0.46, 1.16, 0.03, 0.05, 0.18, 0.95, 0.06);

X.full[,3] = c(1.06, 5.74, 0.27, 0.83, 0.07, 0.07, 2.12, 2.89,0.76, 0.07, 0.50, 0.59, 0.40, 0.28, 0.49, 0.23,0.50, 0.25, 0.08, 0.04, 0.59, 0.24, 0.21, 0.43,0.29, 0.43, 0.23, 2.70, 0.66, 0.49, 0.49, 0.18,0.99, 1.47, 1.82, 0.08, 0.14, 0.28, 0.41, 0.18);

X.full[,4] = c(0.5326, 3.6183, 0.2594, 1.0346, 0.0381, 0.3440, 1.4559, 4.0182,

0.4600, 0.1540, 0.6516, 0.0611, 0.1922, 0.0931, 0.0538, 0.0199,

0.0419, 0.1093, 0.0328, 0.0797, 0.1855, 0.1572, 0.0998, 0.2804,

0.2879, 0.6810, 0.3242, 2.6013, 0.4469, 0.2436, 0.4400, 0.3351,

1.3979, 2.0138, 1.9356, 0.1050, 0.2207, 0.0180, 0.1017, 0.0962);

X.full[1:8,]=20

X= cbind(1L,X.full)

ASM = function(k.s,intcpt = TRUE){

if (intcpt == FALSE){p = p+1}

A = NULL

for(i in 1:p){

A0 = cbind(0L,A)

A1 = cbind(1L,A)

A = rbind(A0,A1)

}# end i-for-loop

if (intcpt == TRUE){

A = cbind(1L,A)

}

return(A[order(apply(A,1,sum)),])

} # end ASM function

A=ASM(p)

p.alpha=apply(A,1,sum)

n.a = dim(A)[1]

m.1=NULL

M = matrix(0,n.a,1)

M.1 = matrix(0,n.a,1)

M.2 = matrix(0,n.a,1)

for(l in 1:L){

set.seed(l)

e=rcauchy(n,location=0,scale=1)

y=X%*%beta+e

nK.corrected = 0

M1=matrix(0,n.a,1)

M2=matrix(0,n.a,1)

Mn=matrix(0,n.a,1)

dat=data.frame(y,X)

Mf.MM = rlm(y~-1+X,data=dat,method="MM",maxit=200)

scale = mad(Mf.MM$res)

r=Mf.MM$res

rank=rank(abs(r))

s1=which(rank<=n/4)

s2=which(rank>n/4 & rank<=2*n/4)

s3=which(rank>2*n/4 & rank<=3*n/4)

s4=which(rank>3*n/4 & rank<=n)

for(k in 1:K){

bs.length = 0

while (bs.length < p+1){

bs = c(sample(s1,m/4,replace=TRUE),sample(s2,m/4,replace=TRUE),sample(s3,m/4,replace=TRUE), sample(s4,m/4,replace=TRUE))

y.st=y[bs]

bs.length=dim(table(bs))

}

for(a in 1:n.a){

X1=as.matrix(X[,A[a,]>0])

X.st=as.matrix(X1[bs,])

lm.fit.a = rlm(y.st~ -1 + X.st,method="MM",maxit=200)

R.b =y[-bs]-as.matrix(X1[-bs,])%*%as.matrix(coef(lm.fit.a),ncol=1)

M2[a]=M2[a]+sum(apply(cbind(R.b^2/(scale^2),b^2),1,min))

} #closing iterating over all models

nK.corrected = nK.corrected+length(R.b)

} #closing iterating over the bootstrap replications

for(a in 1:n.a){

X1=as.matrix(X[,A[a,]>0])

lm.fit.a = rlm(y~ -1 + X1,method="MM",maxit=100)

M1[a] = 1/n*(sum(apply(cbind(lm.fit.a$res^2/(scale^2),b^2),1,min)) +log(n)*p.alpha[a])

Mn[a]=scale^2*(M1[a]+M2[a]/nK.corrected)

} #closing the n.a models to be tested

M[which.min(Mn)]=M[which.min(Mn)]+1

M.2[which.min(M2)]=M.2[which.min(M2)]+1

M.1[which.min(M1)]=M.1[which.min(M1)]+1

} #closing the L loops to be run

m.1=cbind(m.1,M,M.1,M.2)

**Appendix B: Data Sets**

## B-1. Solid waste data of Gunst and Mason (1980)

| S.No | X_1_ | X_2_ | X_3_ | X_4_ |  |
| --- | --- | --- | --- | --- | --- |
| 1 | 0.36 | 0.53 | 1.06 | 0.5326 |  |
|  | 2 | 1.32 | 2.52 | 5.74 | 3.6183 |
|  | 3 | 0.06 | 0.09 | 0.27 | 0.2594 |
|  | 4 | 0.16 | 0.41 | 0.83 | 1.0346 |
|  | 5 | 0.01 | 0.02 | 0.07 | 0.0381 |
|  | 6 | 0.02 | 0.07 | 0.07 | 0.3440 |
|  | 7 | 0.56 | 0.62 | 2.12 | 1.4559 |
|  | 8 | 0.98 | 1.06 | 2.89 | 4.0182 |
|  | 9 | 0.32 | 0.20 | 0.76 | 0.4600 |
|  | 10 | 0.01 | 0.00 | 0.07 | 0.1540 |
|  | 11 | 0.15 | 0.25 | 0.50 | 0.6516 |
|  | 12 | 0.24 | 0.28 | 0.59 | 0.0611 |
|  | 13 | 0.11 | 0.35 | 0.40 | 0.1922 |
|  | 14 | 0.08 | 0.13 | 0.28 | 0.0931 |
|  | 15 | 0.61 | 0.85 | 0.49 | 0.0538 |
|  | 16 | 0.03 | 0.03 | 0.23 | 0.0199 |
|  | 17 | 0.06 | 0.11 | 0.50 | 0.0419 |
|  | 18 | 0.02 | 0.08 | 0.25 | 0.1093 |
|  | 19 | 0.04 | 0.24 | 0.08 | 0.0328 |
|  | 20 | 0.00 | 0.02 | 0.04 | 0.0797 |
|  | 21 | 0.09 | 0.18 | 0.59 | 0.1855 |
|  | 22 | 0.02 | 0.16 | 0.24 | 0.1572 |
|  | 23 | 0.02 | 0.11 | 0.21 | 0.0998 |
|  | 24 | 0.05 | 0.24 | 0.43 | 0.2804 |
|  | 25 | 0.11 | 0.39 | 0.29 | 0.2879 |
|  | 26 | 0.18 | 0.11 | 0.43 | 0.6810 |
|  | 27 | 0.04 | 0.09 | 0.23 | 0.3242 |
|  | 28 | 0.85 | 1.33 | 2.70 | 2.6013 |
|  | 29 | 0.17 | 0.32 | 0.66 | 0.4469 |
|  | 30 | 0.08 | 0.12 | 0.49 | 0.2436 |
|  | 31 | 0.38 | 0.18 | 0.49 | 0.4400 |
|  | 32 | 0.11 | 0.13 | 0.18 | 0.3351 |
|  | 33 | 0.39 | 0.38 | 0.99 | 1.3979 |
|  | 34 | 0.43 | 0.46 | 1.47 | 2.0138 |
|  | 35 | 0.57 | 1.16 | 1.82 | 1.9356 |
|  | 36 | 0.13 | 0.03 | 0.08 | 0.1050 |
|  | 37 | 0.04 | 0.05 | 0.14 | 0.2207 |
|  | 38 | 0.13 | 0.18 | 0.28 | 0.0180 |
|  | 39 | 0.20 | 0.95 | 0.41 | 0.1017 |
|  | 40 | 0.07 | 0.06 | 0.18 | 0.0962 |

**Source: Gunst, R. F., & Mason, R. L. (1980). *Regression Analysis and its Applications*, New York: Marcel Dekker.**

## B-2. Stack Loss Data

| S.No. | X_1_ |  | X_2_ |  | X_3_ |  | Y |
| --- | --- | --- | --- | --- | --- | --- | --- |
| 1 | 80.00 |  | 27.00 |  | 89.00 |  | 42.00 |
| 2 | 80.00 |  | 27.00 |  | 88.00 |  | 37.00 |
| 3 | 75.00 |  | 25.00 |  | 90.00 |  | 37.00 |
| 4 | 62.00 |  | 24.00 |  | 87.00 |  | 28.00 |
| 5 | 62.00 |  | 22.00 |  | 87.00 |  | 18.00 |
| 6 | 62.00 |  | 23.00 |  | 87.00 |  | 18.00 |
| 7 | 62.00 |  | 24.00 |  | 93.00 |  | 19.00 |
| 8 | 62.00 |  | 24.00 |  | 93.00 |  | 20.00 |
| 9 | 58.00 |  | 23.00 |  | 87.00 |  | 15.00 |
| 10 | 58.00 |  | 18.00 |  | 80.00 |  | 14.00 |
| 11 | 58.00 |  | 18.00 |  | 89.00 |  | 14.00 |
| 12 | 58.00 |  | 17.00 |  | 88.00 |  | 13.00 |
| 13 | 58.00 |  | 18.00 |  | 82.00 |  | 11.00 |
| 14 | 58.00 |  | 19.00 |  | 93.00 |  | 12.00 |
| 15 | 50.00 |  | 18.00 |  | 89.00 |  | 8.00 |
| 16 | 50.00 |  | 18.00 |  | 86.00 |  | 7.00 |
| 17 | 50.00 |  | 19.00 |  | 72.00 |  | 8.00 |
| 18 | 50.00 |  | 19.00 |  | 79.00 |  | 8.00 |
| 19 | 50.00 |  | 20.00 |  | 80.00 |  | 9.00 |
| 20 | 56.00 |  | 20.00 |  | 82.00 |  | 15.00 |
| 21 | 70.00 |  | 20.00 |  | 91.00 |  | 15.00 |

**Source: Bro K. A. (1965). *Statistical Theory and Methodology in Science an d Engineering*. Wiley, New York.**
